# Reproductive tract herpes-like lesions associated with bovine herpesvirus 4 in dairy cows

**DOI:** 10.1128/asmcr.00055-26

**Published:** 2026-09-22

**Authors:** Jianwei Zhang, Yuxin Lai, Hanyu Lin, Hongzuo Duan, Yiping Gu, Guangjun Chang, Chunlin Han, Zihao Pan

**Affiliations:** 1 OIE Reference Laboratory for Swine Streptococcosis, College of Veterinary Medicine, Nanjing Agricultural University 70578, Nanjing, China; 2 College of Veterinary Medicine, Nanjing Agricultural University 70578, Nanjing, China; 3 Technical Support Center, Modern Farming (Group) Co. Ltd., Anhui, China; Pattern Bioscience, Austin, Texas, USA

**Keywords:** physiological stage, dairy cows, reproductive tract herpes-like lesions, bovine herpesvirus 4

## Abstract

Reproductive tract herpes-like lesions have been sporadically observed in dairy cows, but their pathological definition and etiological relevance remain unclear. In China, bovine herpesvirus 4 (BoHV-4) has recently gained attention as a pathogen associated with reproductive disorders in dairy cattle, including vulvovaginal herpes-like lesions and reproductive tract hemorrhage. In this case series, dairy cows presenting with a distinct clinical phenotype characterized by reproductive tract herpes-like lesions were investigated across different physiological stages. While herpes-like lesions were observed in all examined groups, hemorrhagic erosions of the reproductive tract mucosa were predominantly identified in early postpartum and dry cows. Pathological, molecular, and virological analyses were performed on lesion-associated tissues. BoHV-4 DNA was predominantly detected in samples from pregnant, dry, and early postpartum cows, whereas tissues from neonatal calves and replacement heifers were consistently negative. Infectious BoHV-4 was successfully isolated from lesion tissues and characterized by immunofluorescence assay and transmission electron microscopy. Histopathological analysis revealed multi-focal epithelial degeneration accompanied by cytoplasmic vacuolation, scattered focal epithelial necrosis, and diffuse inflammatory infiltration within the lamina propria, and immunohistochemistry demonstrated BoHV-4 antigen localization in lesion-associated epithelial and subepithelial regions. Although definitive causality cannot be established, the combined molecular, virological, and pathological findings support an association between BoHV-4 infection and this reproductive tract herpes-like clinical phenotype, which may represent a previously underdefined clinical-pathological presentation in dairy cows, particularly during specific physiological stages.

## INTRODUCTION

Bovine herpesvirus 4 (BoHV-4) is a member of the Gammaherpesvirinae and has been associated with a broad spectrum of inflammatory and reproductive disorders in cattle (1–3), including metritis, vaginitis, endometritis, abortion, and other conditions involving the reproductive tract. Although BoHV-4 is widely recognized as a virus capable of establishing latent or persistent infection (4), its precise role in naturally occurring disease remains incompletely understood, and clinicopathological presentations linked to infection are often poorly defined.

In recent years, BoHV-4 has gained increasing attention in China as a pathogen potentially relevant to reproductive disease in dairy cattle. Clinical observations in affected herds have suggested an association between BoHV-4 infection and reproductive tract abnormalities, including vulvovaginal herpes-like lesions, mucosal hemorrhage, and postpartum reproductive disorders. However, most available reports remain limited to molecular detection or virus isolation, while systematic descriptions integrating clinical appearance, histopathology, immunohistochemistry, and virological evidence are still scarce. Therefore, the clinical and pathological features of BoHV-4-associated reproductive tract lesions remain insufficiently defined.

In the present case series, we investigated dairy cows from a commercial Holstein herd that exhibited a reproducible clinical presentation characterized by herpes-like lesions of the reproductive tract across different physiological stages. Because the gross manifestations were not identical among affected groups, particular attention was paid to differences between age and physiological categories, especially the occurrence of hemorrhagic erosions in specific stages. By combining pathological, molecular, and virological analyses, this study aimed to characterize this underdefined reproductive tract clinical phenotype and to evaluate its potential association with BoHV-4 infection. To our knowledge, this is the first case series to systematically characterize this reproductive tract herpes-like clinical presentation across different physiological stages in dairy cows using combined pathological, molecular, and virological approaches.

## MATERIALS AND METHODS

### Herd information and case enrollment

The cases described in this study were derived from a large‑scale commercial Holstein dairy herd with approximately 12,000 cattle in China. During routine herd monitoring, farm veterinarians identified a widespread outbreak of atypical vulvar and vaginal mucosal lesions across cattle at all physiological stages. These lesions exhibited distinct herpes-like morphological features, and some affected individuals additionally presented obvious mucosal hemorrhage and erosion, which prompted our systematic field investigation.

A total of 25 symptomatic cattle were enrolled in this case series. In consideration of animal welfare principles and the farm’s production management regulations, we selected only five clinically affected animals with typical herpes-like reproductive tract lesions from each of five physiological subgroups: neonatal calves, replacement heifers, pregnant cows, dry cows, and early postpartum cows (within 15 days after calving). This stratified sampling design was adopted to enable comparative analysis of lesion characteristics among different developmental and reproductive stages. All enrolled animals displayed apparent genital lesions upon clinical inspection, while none presented with concurrent dermatitis-related cutaneous lesions at the time of sampling.

The absence of dermatitis-related skin lesions differentiates these cases from previously reported bovine syndromes with concurrent dermatitis and hemorrhage. Information regarding systemic clinical abnormalities, reproductive performance, recent herd management changes, dietary changes, animal introductions, and therapeutic interventions was reviewed where available. No major recent feed changes or new animal introductions were documented during the observation period. No specific antimicrobial or antiviral treatment had been initiated prior to sample collection. Milk production data showed no consistent decline among affected animals. Because the affected animals were identified under field conditions in a commercial herd, the cases were interpreted as naturally occurring rather than experimentally induced disease.

### Sample collection

Vaginal swab specimens and tissues associated with visible lesions were aseptically obtained from each animal. Lesion mucosal tissues were collected via clinical biopsy from live cattle, and no necropsy sampling was performed in this field case series. All vaginal swabs and biopsy tissues were collected aseptically from vulvar and vaginal lesion sites during routine farm veterinary examinations. The collected tissues were divided and allocated for molecular analysis, virus isolation, and histopathological evaluation. Specimens designated for histological assessment were fixed in 10% neutral-buffered formalin, whereas samples intended for virological assays were either processed immediately or stored at −80°C prior to analysis.

### Molecular detection of BoHV-4

Genomic DNA was extracted from vaginal swab specimens and lesion-associated tissues using a commercially available extraction kit (Vazyme, Nanjing, China) according to the manufacturer’s instructions. Detection of BoHV-4 was performed using a dual-fluorescence real-time quantitative PCR kit (Qingdao Lijian Biotechnology Co., Ltd., Qingdao, China) targeting the glycoprotein L (gL) gene (5). The primer/probe set used for BoHV-4 detection corresponded to the BoHV-4 reference genome NC_002665.1. Reactions were performed in a total volume of 25 μL, consisting of 20 μL of reaction mixture and 5 μL of extracted DNA. Samples yielding a cycle threshold (Ct) value of ≤38 were considered positive.

### Virus isolation and immunofluorescence assay

Homogenates prepared from lesion-associated tissues were inoculated onto confluent monolayers of Madin–Darby bovine kidney (MDBK) cells for virus isolation (6). Cultures were monitored daily for cytopathic effects, and affected cultures were further passaged to confirm viral replication. An indirect immunofluorescence assay was performed using a fluorescein isothiocyanate (FITC)-conjugated monoclonal antibody specific for BoHV-4 (Bio-X Diagnostics, Rochefort, Belgium; catalog no. BIO 286) at a dilution of 1:500.

### Transmission electron microscopy

MDBK cells exhibiting virus-induced cytopathic changes were processed for transmission electron microscopy using standard procedures. Ultrathin sections were examined with a transmission electron microscope (HT7700; Hitachi, Japan).

### Histopathology and immunohistochemistry

Histopathological evaluation was performed on lesion-associated reproductive tract tissues using routine hematoxylin and eosin (HE) staining. For immunohistochemical analysis, formalin-fixed, paraffin-embedded tissue sections were processed using standard protocols, including deparaffinization, rehydration, and antigen retrieval. Sections were incubated with the same monoclonal antibody against BoHV-4 used for immunofluorescence analysis (Bio-X Diagnostics, Rochefort, Belgium; catalog no. BIO 286) at a dilution of 1:500, optimized for tissue sections. Immunoreactivity was visualized using a standardized chromogenic detection system, and negative controls were included in each staining run.

## RESULTS

### Gross clinical lesion characteristics

**Fig 1:**
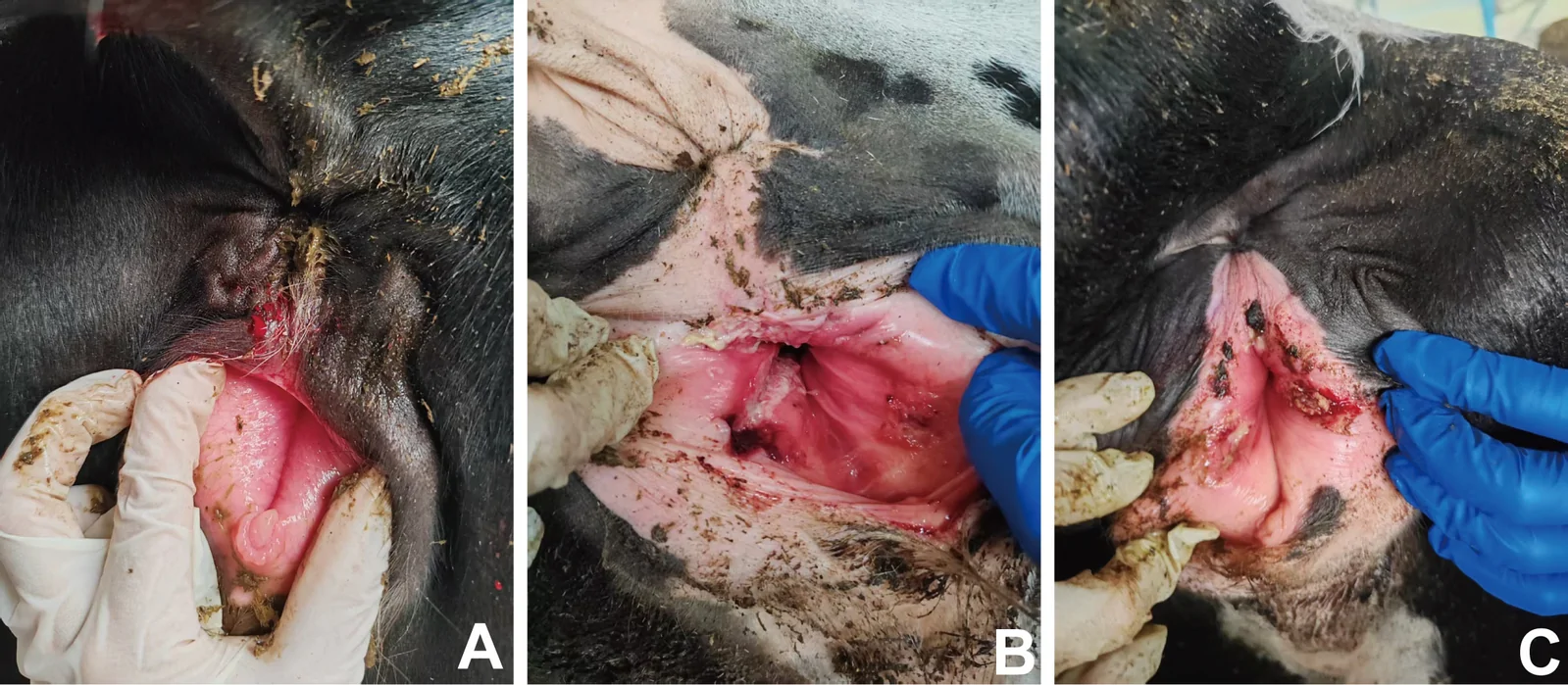
Gross appearance of reproductive tract herpes-like lesions in dairy cows. Gross appearance of herpes-like lesions on the vulvar and vaginal mucosa of dairy cows. Lesions were characterized by raised, vesicular, or plaque-like changes. In early postpartum and dry cows, the vaginal mucosa exhibited variable degrees of hemorrhagic erosion. (**A**) Representative raised, vesicular, or plaque-like lesions on the vaginal mucosa. (**B and C**) Representative hemorrhagic and erosive lesions of the vaginal mucosa showing variable degrees of mucosal bleeding and erosion.

Reproductive tract herpes-like lesions were identified in affected cattle across all examined physiological groups, including neonatal calves, replacement heifers, pregnant cows, early postpartum cows, and dry cows. Grossly, the lesions were consistently localized to the vulvar and vaginal mucosa and were characterized by raised, vesicular, or plaque-like changes. In contrast, hemorrhagic erosions were not uniformly distributed among groups but were predominantly observed in early postpartum and dry cows, in which hemorrhagic changes of the vulvar or vaginal mucosa were more frequently documented (Fig. 1). No cutaneous lesions consistent with dermatitis were observed in any of the examined animals.

### Molecular detection of BoHV-4

BoHV-4 DNA was detected predominantly in lesion-associated tissues and vaginal swabs obtained from pregnant cows, dry cows, and early postpartum cows, whereas all samples collected from neonatal calves and replacement heifers were negative (Table 1). Specifically, BoHV-4 DNA was detected in 3/5 vaginal tissue samples and 3/5 vaginal swabs from pregnant cows, 1/5 vaginal tissue samples and 1/5 vaginal swabs from early postpartum cows, and 5/5 vaginal tissue samples and 5/5 vaginal swabs from dry cows. In contrast, BoHV-1 DNA was not detected in any tissue or swab sample examined.

**TABLE 1:** Summary of virological and immunohistochemical findings in dairy cows at different physiological stages

Physiological stage	*n*	qPCR (tissue)	qPCR (swab)	Virus isolation	IHC positivity	IFA-positive isolates^*a*^
Neonatal calves	5	0/5	0/5	0/5	2/5	NA
Replacement heifers	5	0/5	0/5	0/5	2/5	NA
Pregnant cows	5	3/5	3/5	2/5	3/5	2/2
Early postpartum cows	5	1/5	1/5	1/5	4/5	1/1
Dry cows	5	5/5	5/5	3/5	5/5	3/3

^*a*^ IFA, indirect immunofluorescence; NA, no isolates available (no virus isolates were recovered in this group, resulting in zero IFA‑positive isolates).

### Virus isolation and identification

**Fig 2:**
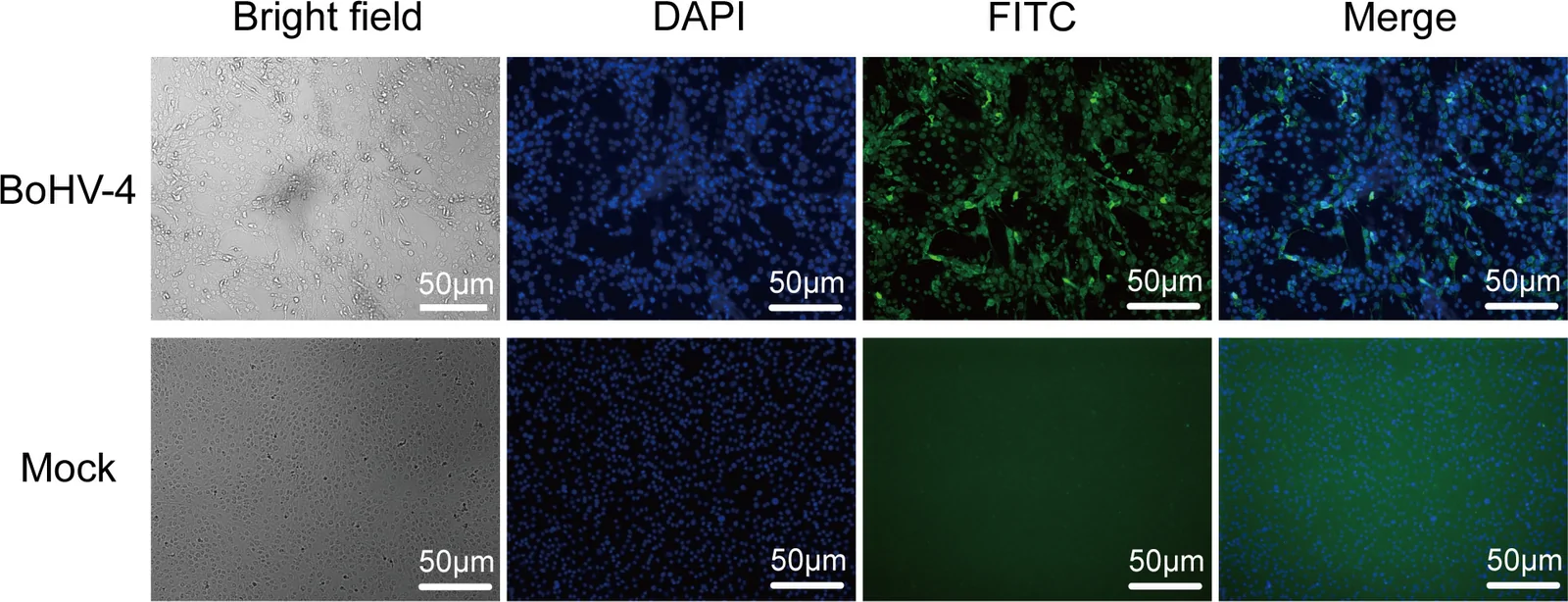
Immunofluorescence detection of bovine herpesvirus 4 in MDBK cells. Detection of BoHV-4 in MDBK cells by indirect immunofluorescence assay. Infected cells exhibited specific green fluorescence following staining with an FITC-labeled monoclonal antibody against BoHV-4. Nuclei were counterstained with 4′,6‑diamidino‑2‑phenylindole (DAPI) (blue). “Merge” represents the overlay of fluorescence and nuclear staining images.

Infectious BoHV-4 was successfully isolated from lesion-associated tissues obtained from BoHV-4-positive cows at different physiological stages. Specifically, two isolates were recovered from pregnant cows, one from an early postpartum cow, and three from dry cows. Following inoculation onto MDBK cells, the isolates induced marked cytopathic effects characterized by cell rounding, detachment, and disruption of monolayer integrity (7). Viral identity was further validated via indirect immunofluorescence, which revealed distinct green fluorescent signals within infected cells (Fig. 2).

### Transmission electron microscopy findings

**Fig 3:**
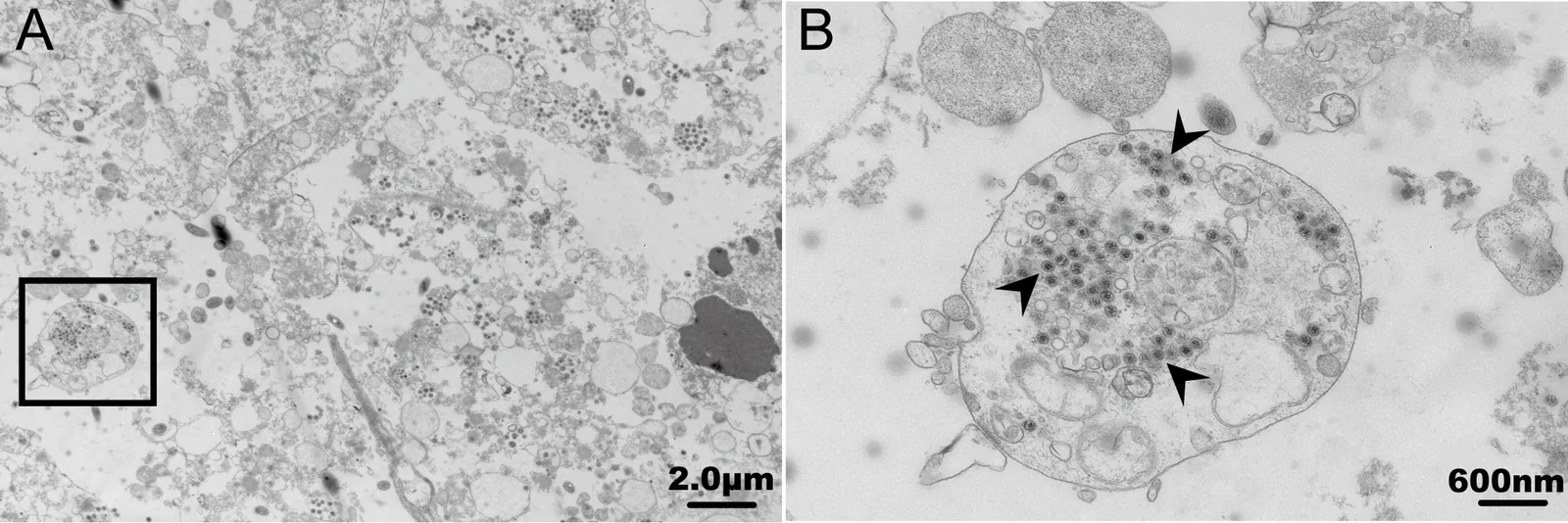
Transmission electron microscopy of bovine herpesvirus 4 in MDBK cells. Transmission electron microscopy of MDBK cells infected with BoHV-4. (**A**) Infected cells showing herpesvirus-like particles. (**B**) Higher magnification of boxed regions demonstrating enveloped viral particles with icosahedral nucleocapsids. Scale bars are indicated in the images.

Enveloped herpesvirus-like particles containing icosahedral nucleocapsids were observed within infected MDBK cells (8), consistent with the ultrastructural morphology of herpesviruses (Fig. 3).

### Histopathological and immunohistochemical findings

**Fig 4:**
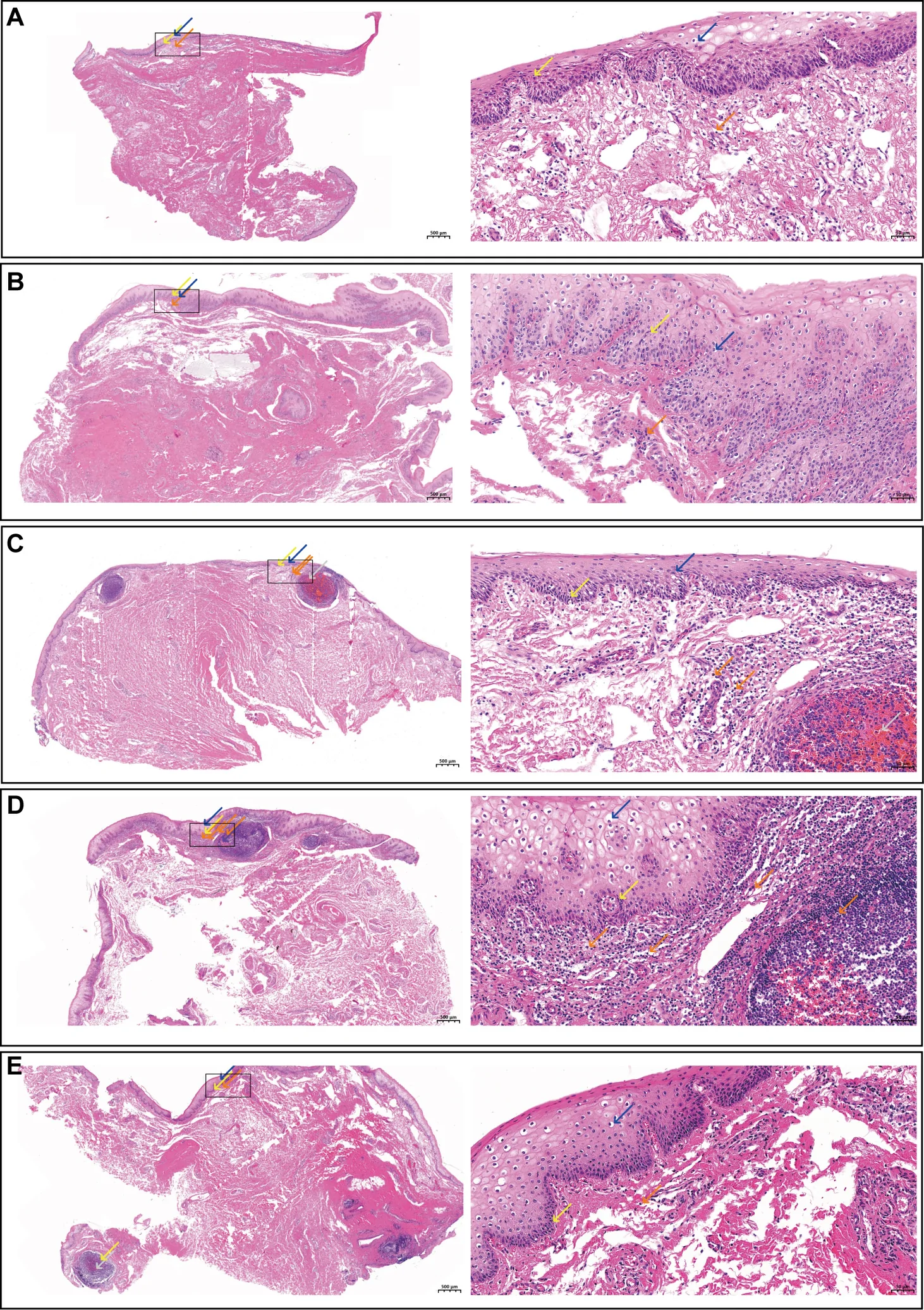
Histopathological features of reproductive tract herpes-like lesions in dairy cows. Histopathological examination of reproductive tract tissues from dairy cows at different physiological stages. The mucosal epithelium showed uneven thickness and consisted of stratified squamous epithelium, with flattened squamous cells in the superficial layer and columnar cells in the deeper layer. Numerous epithelial cells exhibited cytoplasmic loosening or vacuolar degeneration (blue arrows), and a small number of epithelial cells showed necrosis with nuclear pyknosis (yellow arrows). The lamina propria consisted of loosely arranged fibrous connective tissue with scattered lymphocytic infiltration (orange arrows). Black boxed areas indicate regions shown at higher magnification. Hematoxylin and eosin staining. (**A**) Neonatal calves. (**B**) Replacement heifers. (**C**) Pregnant cows. (**D**) Early postpartum cows. (**E**) Dry cows.

**Fig 5:**
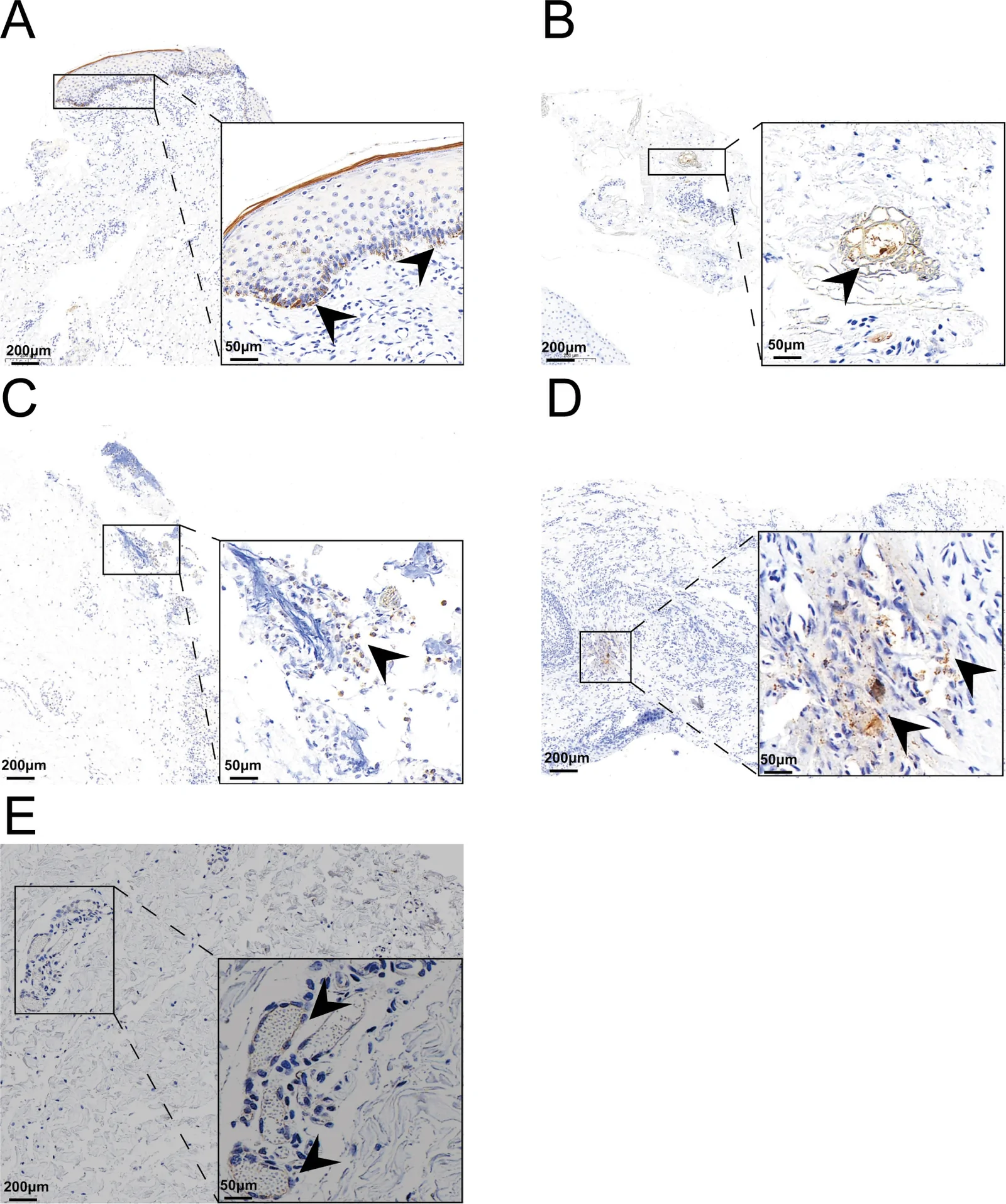
Immunohistochemical detection of bovine herpesvirus 4 antigen in reproductive tract tissues. Immunohistochemical staining for BoHV-4 antigen in reproductive tract tissues from dairy cows. Brown signals represent BoHV-4-specific immunoreactivity, which primarily accumulates within the cytoplasm of lesional mucosal epithelial cells, with weak signals visible in partial subepithelial inflammatory cells. Black arrows indicate representative positively stained regions. (**A**) Neonatal calves. (**B**) Replacement heifers. (**C**) Pregnant cows. (**D**) Early postpartum cows. (**E**) Dry cows.

Microscopic lesions were observed in both BoHV-4 DNA-positive and BoHV-4 DNA-negative tissues. In BoHV-4 DNA-positive tissues, multi-focal epithelial degeneration accompanied by prominent cytoplasmic vacuolation was the dominant microscopic lesion, together with scattered focal epithelial necrosis. The lamina propria exhibited diffuse inflammatory infiltration mainly composed of lymphocytes with sporadic plasma cells (Fig. 4). In these lesion-bearing tissues, immunohistochemistry showed BoHV-4 antigens predominantly accumulated within the cytoplasm of damaged mucosal epithelial cells, with minor positive signals detected in subepithelial stromal inflammatory cells (9) (Fig. 5).

By comparison, tissues from neonatal calves and replacement heifers were negative for BoHV-4 DNA by quantitative PCR, and infectious virus could not be recovered. Nevertheless, similar microscopic changes, including epithelial degeneration and mild inflammatory infiltration, were still observed (Fig. 4). In addition, BoHV-4 antigen was detected by immunohistochemistry in epithelial and subepithelial compartments of these PCR-negative tissues (Fig. 5). In contrast, successful virus isolation was achieved only from tissues positive for BoHV-4 DNA, providing additional virological support for viral involvement in those cases.

Taken together, these observations indicate that BoHV-4 antigen may persist in tissues despite the absence of detectable viral DNA, potentially reflecting low-level viral persistence or prior infection rather than active viral replication at the time of sampling.

## DISCUSSION

This case series carries four distinct novel contributions that fill clear gaps in global BoHV-4 literature, especially for Chinese dairy production systems. First, BoHV-4 is an emerging pathogen in mainland China, with its first domestic isolation reported in 2019; systematic multi-stage, clinical-pathological case series describing large-scale field outbreaks of genital herpes-like lesions remain extremely scarce in domestic and international publications from China. Most prior Chinese reports merely performed single molecular detection or sporadic virus isolation without integrated clinical and pathological evidence. Second, this study originates from a 12,000-head commercial Holstein dairy farm experiencing a herd-wide outbreak of uniform vulvar herpes-like mucosal lesions across all physiological groups, a unique field epidemic scenario rarely documented overseas, where BoHV-4 cases are mostly scattered sporadic individuals rather than group outbreaks. Third, we implemented stratified sampling, covering neonatal calves, replacement heifers, pregnant cows, dry cows, and early postpartum cows, and, for the first time, systematically compared stage-dependent differences in gross hemorrhagic lesions, viral PCR positivity, virus isolation recovery, and immunohistochemistry (IHC) antigen retention. We identified a previously unreported PCR-negative but IHC-positive persistent infection phenotype in young cattle groups, which provides new morphological evidence for latent BoHV-4 persistence in juvenile hosts. Fourth, most overseas publications only adopt a single detection technique, while this study combines gross clinical observation, field epidemiological surveys, quantitative PCR (qPCR), virus isolation, transmission electron microscopy, histopathology, and subcellular-targeted IHC. Such multi-dimensional data sets fully characterize this unique genital lesion phenotype, which is rarely documented globally. Collectively, these original field observations and integrated laboratory findings deliver valuable regional epidemiological and pathological data for BoHV-4, an understudied emerging pathogen in Chinese dairy herds, and provide practical diagnostic references for global veterinary clinicians encountering similar genital lesion syndromes.

This case series presents a unique herd-wide outbreak of BoHV-4-associated reproductive tract herpes-like lesions in a large-scale Chinese dairy farm, representing the first systematic multi-physiological-stage case series of this distinct genital lesion syndrome in China, an emerging endemic region for BoHV-4 infection. While scattered BoHV-4 reproductive case reports exist in European and North American literature, nearly all focus on isolated metritis, abortion, or dermatitis-hemorrhagic syndrome (10, 11); herd-wide vulvar herpes-like mucosal lesions spanning calves to postpartum cows have not been comprehensively described. Our stratified comparative analysis further reveals stage-specific viral activity and lesion manifestations, alongside a novel PCR-negative/IHC-positive latent infection pattern, substantially expanding the recognized clinicopathological spectrum of BoHV-4 infection in dairy cattle.

This work further characterizes this consistent herpes-like genital phenotype and integrates multi-layer laboratory evidence to support a causal association with BoHV-4. In the present cases, reproductive tract herpes-like lesions were observed across all examined physiological groups, whereas hemorrhagic erosions were predominantly documented in early postpartum and dry cows. This stage-associated difference in gross appearance suggests that the clinical phenotype was not uniformly expressed among affected animals and may be influenced by host physiological status.

BoHV-4 is a member of the Gammaherpesvirinae (12), a group of herpesviruses characterized by their ability to establish latent or persistent infection in the host (13, 14). Studies have shown that BoHV-4 may persist in cattle and reactivate under conditions of physiological or immunological stress. In dairy cattle, the periparturient period and the onset of lactation are frequently accompanied by transient immune modulation, metabolic stress, and increased susceptibility to reproductive tract disease (15). In this context, the restriction of BoHV-4 DNA detection and successful virus isolation to pregnant, dry, and early postpartum cows in the present series supports the possibility that viral activity may be enhanced during specific physiological stages. Thus, the distribution of virological positivity observed here is consistent with the concept of latent or low-level persistent BoHV-4 infection with stage-associated reactivation (16). A further biologically plausible explanation is that BoHV-4 may establish latent infection in neural tissues associated with the lumbosacral region, with subsequent reactivation leading to viral spread to the reproductive tract. Although this mechanism was not directly investigated in the present study, it is consistent with previous experimental observations and may help explain the localization of lesions to the vulvovaginal mucosa.

An additional important finding was the presence of histopathological lesions and BoHV-4 antigen localization in some tissues, in which viral DNA was not detected by quantitative PCR and infectious virus was not recovered. This discrepancy likely reflects the complex biology of herpesvirus infection rather than a simple inconsistency between methods. Negative molecular results may occur when viral load is low, when viral genomes are distributed focally within tissue, or when sampling does not capture the most actively infected region. At the same time, viral proteins may remain detectable in affected tissues by immunohistochemistry even when amplifiable nucleic acid is absent (17, 18). Therefore, the PCR-negative/IHC-positive pattern observed in neonatal calves and replacement heifers may be compatible with low-level viral persistence, prior infection, or localized antigen retention rather than active productive infection at the time of sampling. The absence of BoHV-1 detection further reduces the likelihood that the observed lesions were associated with other common bovine herpesviruses.

The histopathological changes observed in this study further support the biological relevance of BoHV-4 in the affected tissues. In BoHV-4 DNA-positive cases, epithelial degeneration, cytoplasmic vacuolar change, focal epithelial necrosis, and inflammatory infiltration of the lamina propria were associated with viral antigen localization and, in some animals, successful virus isolation. This spatial and virological concordance was stronger than that observed in BoHV-4 DNA-negative tissues and provides additional support for viral involvement in lesion development. Nevertheless, the detection of comparable yet virologically weaker lesions in PCR-negative animals further indicates that bovine genital mucosal lesions have multi-factorial origins and may be influenced by local inflammation, hormonal variation, tissue injury, or other concurrent factors.

The clinical presentation described here differs in important respects from previously reported bovine syndromes characterized by concurrent dermatitis and hemorrhagic manifestations (19), as no cutaneous lesions compatible with dermatitis were observed in the present animals. Instead, the lesions were confined to the reproductive tract mucosa, supporting the interpretation that these cases represent a distinct reproductive tract clinical phenotype rather than a broader multi-system syndrome. This distinction may be relevant for field diagnosis, particularly in commercial dairy herds where localized vulvovaginal lesions might otherwise be overlooked or attributed to nonspecific trauma or postpartum injury.

From a herd health perspective, the findings also suggest that BoHV-4 should be considered in the differential diagnosis of vulvovaginal herpes-like lesions and reproductive tract hemorrhagic erosions, particularly in cows during the dry and early postpartum periods. Because BoHV-4 is believed to spread through close contact with infected secretions and may persist latently in infected animals, the presence of clinically affected cows in different physiological groups may suggest the possibility of viral circulation within the herd. Although this study was not designed to define transmission pathways, the findings emphasize the importance of continued herd observation, targeted sampling of lesion-associated tissues, and integration of molecular testing with pathology and immunohistochemistry in similar field cases.

Several limitations should be acknowledged. The number of animals in each physiological group was limited, the observations were obtained under natural field conditions, and herd-level production and reproductive performance data were not comprehensively evaluated. In addition, although the combined evidence strongly supports an association between BoHV-4 and the lesions described, the observational design does not permit definitive conclusions regarding causation. Even so, the present series expands the recognized range of pathological manifestations potentially associated with BoHV-4 in dairy cows and supports the view that reproductive tract herpes-like lesions, particularly when accompanied by hemorrhagic erosion in early postpartum and dry cows, may represent a poorly characterized clinical-pathological presentation linked to BoHV-4 infection.

## Data Availability

The data that support the findings of this study are available from the corresponding author upon reasonable request.

## References

[1] Donofrio G, Herath S, Sartori C, Cavirani S, Flammini CF, Sheldon IM. 2007. Bovine herpesvirus 4 is tropic for bovine endometrial cells and modulates endocrine function. Reproduction 134:183–197. doi:10.1530/REP-07-0065 PMC2740819 17641100

[2] Areda D, Chigerwe M, Crossley B. 2018. Bovine herpes virus type-4 infection among postpartum dairy cows in California: risk factors and phylogenetic analysis. Epidemiol Infect 146:904–912. doi:10.1017/S0950268818000791 PMC9184933 29633683

[3] Yang B, Li Y, Bogado Pascottini O, Xie J, Wei R, Opsomer G, Nauwynck H. 2017. Primary replication and invasion of the bovine gammaherpesvirus BoHV-4 in the genital mucosae. Vet Res 48:83. doi:10.1186/s13567-017-0489-3 PMC5706299 29183401

[4] Bauermann FV, Falkenberg SM, Martins M, Dassanayake RP, Neill JD, Ridpath JF, Silveira S, Palmer MV, Buysse A, Mohr A, Flores EF, Diel DG. 2022. Genome sequence and experimental infection of calves with bovine gammaherpesvirus 4 (BoHV-4). Arch Virol 167:1659–1668. doi:10.1007/s00705-022-05486-8 35708765

[5] Romeo F, Spetter MJ, Moran P, Pereyra S, Odeon A, Perez SE, Verna AE. 2020. Analysis of the transcripts encoding for antigenic proteins of bovine gammaherpesvirus 4. J Vet Sci 21:e5. doi:10.4142/jvs.2020.21.e5 PMC7000896 31940684

[6] Kruger ER, Penha TR, Stoffelo DRE, Roehe PM, Ribeiro MC, Soccol VT. 2015. Bovine Herpesvirus 4 in Parana State, Brazil: case report, viral isolation, and molecular identification. Braz J Microbiol 46:279–283. doi:10.1590/S1517-838246120130949 PMC4512082 26221118

[7] Morán PE, Pérez SE, Odeón AC, Verna AE. 2015. Herpesvirus bovino 4 (BoHV-4): aspectos generales de su biología y situación en la República Argentina. Rev Argent Microbiol 47:155–166. doi:10.1016/j.ram.2015.02.007 25962539

[8] Lin J, Chen R-H, Yang M-J, Zhu Y-M, Xue F. 2021. Isolation and molecular characterization of bovine herpesvirus 4 from cattle in mainland China. Arch Virol 166:619–626. doi:10.1007/s00705-020-04896-w PMC7788162 33410994

[9] Zhu L, Dai X, Lai Y, Guo Z, Gu Y, Zhang J, Wang H, Chang G, Yao H, Pan Z. 2024. Unveiling the role of bovine herpesvirus type 4 (BHV-4) in dairy cow reproductive disorders: insights from a multifaceted study in China. Transbound Emerg Dis 2024:4048149. doi:10.1155/tbed/4048149 PMC12016887 40303107

[10] Frazier K, Pence M, Mauel MJ, Liggett A, Hines ME, Sangster L, Lehmkuhl HD, Miller D, Styer E, West J, Baldwin CA. 2001. Endometritis in Postparturient Cattle Associated with Bovine Herpesvirus-4 Infection: 15 Cases. J Vet Diagn Invest 13:502–508. doi:10.1177/104063870101300608 11724141

[11] Delooz L, Czaplicki G, Houtain JY, Dal Pozzo F, Saegerman C. 2017. Laboratory Findings Suggesting an Association Between BoHV-4 and Bovine Abortions in Southern Belgium. Transbound Emerg Dis 64:1100–1109. doi:10.1111/tbed.12469 26752765

[12] Zimmermann W, Broll H, Ehlers B, Buhk HJ, Rosenthal A, Goltz M. 2001. Genome sequence of bovine herpesvirus 4, a bovine *Rhadinovirus*, and identification of an origin of DNA replication. J Virol 75:1186–1194. doi:10.1128/JVI.75.3.1186-1194.2001 PMC114024 11152491

[13] Castrucci G, Frigeri F, Ferrari M, Pedini B, Aldrovandi V, Cilli V, Rampichini L, Gatti R. 1987. Reactivation in calves of latent infection by Bovid herpesvirus-4. Microbiologica 10:37–45. 3033450

[14] Dubuisson J, Thiry E, Bublot M, Thomas I, van Bressem MF, Coignoul F, Pastoret PP. 1989. Experimental infection of bulls with a genital isolate of bovine herpesvirus-4 and reactivation of latent virus with dexamethasone. Vet Microbiol 21:97–114. doi:10.1016/0378-1135(89)90022-9 2558442

[15] Sheldon IM, Williams EJ, Miller ANA, Nash DM, Herath S. 2008. Uterine diseases in cattle after parturition. Vet J 176:115–121. doi:10.1016/j.tvjl.2007.12.031 PMC2706386 18329302

[16] Donofrio G, van Santen VL. 2001. A bovine macrophage cell line supports bovine herpesvirus-4 persistent infection. J Gen Virol 82:1181–1185. doi:10.1099/0022-1317-82-5-1181 11297693

[17] Campos FS, Franco AC, Oliveira MT, Firpo R, Strelczuk G, Fontoura FE, Kulmann MIR, Maidana S, Romera SA, Spilki FR, Silva AD, Hübner SO, Roehe PM. 2014. Detection of bovine herpesvirus 2 and bovine herpesvirus 4 DNA in trigeminal ganglia of naturally infected cattle by polymerase chain reaction. Vet Microbiol 171:182–188. doi:10.1016/j.vetmic.2014.03.012 24725448

[18] Deim Z, Szeredi L, Tompó V, Egyed L. 2006. Detection of bovine herpesvirus 4 in aborted bovine placentas. Microb Pathog 41:144–148. doi:10.1016/j.micpath.2006.03.006 16950594

[19] Bellino C, Iussich S, Biasato I, Peletto S, Caruso C, Gianella P, Cagnasso A, D’Angelo A. 2015. Potential pathogenetic role of bovine herpesvirus 4 in two dairy cows with dermatitis-pyrexia-hemorrhagic syndrome. J Clin Microbiol 53:2763–2767. doi:10.1128/JCM.00717-15 PMC4508391 26041898

